# The Relationship Between Falling Distance and Trauma Severity Among Fall Injury Survivors Who Were Transported to a Trauma Center

**DOI:** 10.7759/cureus.25099

**Published:** 2022-05-18

**Authors:** Kyoko Muneshige, Masayuki Miyagi, Gen Inoue, Toshiyuki Nakazawa, Takayuki Imura, Terumasa Matsuura, Tadashi Kawamura, Yuichi Kataoka, Yasushi Asari, Masashi Takaso

**Affiliations:** 1 Department of Orthopedic Surgery, Kitasato University School of Medicine, Sagamihara, JPN; 2 Department of Emergency and Critical Care Medicine, Kitasato University School of Medicine, Sagamihara, JPN

**Keywords:** mccormack load-sharing classification, fall injury, vertebral fracture, hospitalization, falling distance

## Abstract

Introduction

Falls from >6 meters have been shown to cause multiple traumas and serious injuries. However, especially among fall survivors who were transported to trauma centers, the relationship between falling distance and trauma severity remains unclear. This study aimed to investigate the relationship between falling distance and trauma severity among fall injury survivors who were transported to a trauma center and clarify the characteristics of trauma among survivors of falls from high places from an orthopedic surgeon’s perspective.

Methods

We retrospectively reviewed the medical records of 65 fall injury survivors who were transported to a trauma center for falling distance; whether the fall was a suicide attempt; abdominal, chest, and head trauma; the numbers of upper-limb, lower-limb, and spinal vertebral fractures; McCormack load-sharing classification score; unstable pelvic fracture; Frankel classification; injury severity score (ISS); and duration of intensive care unit (ICU) and hospital stay. We evaluated the correlations between falling distance and the other factors and compared all factors between those falling <6 meters and those falling >6 meters.

Results

Falling distance was weakly positively correlated with durations of ICU and hospital stay. The percentage of cases that were suicide attempts, the number of lower-limb fractures, the McCormack load-sharing classification score, and the durations of ICU and hospital stay were significantly higher among those falling from >6 meters than among those falling from <6 meters. Conversely, there were no significant differences in abdominal trauma, chest trauma, head trauma, number of upper-limb fractures, number of vertebral fractures, unstable pelvic fracture, or Frankel classification between the two groups.

Conclusion

The findings indicate that falling from a higher distance may increase lower-limb and vertebral fracture severity and may lead to longer ICU and hospital stays among fall injury survivors who are transported to trauma centers.

## Introduction

Fall from height injuries are high-energy traumas caused by rapid deceleration. They cause multiple fractures, organ damage, and severe injury. Several authors have identified factors that determine the severity of a fall injury, including falling distance and ground characteristics where the fall occurs [1,2]. Sasser et al. have recommended transporting patients with fall injuries after falling from a height of at least 20 feet (approximately six meters) to a trauma center because of the likely severity of their injuries [3]. Additionally, Frederic et al. reported a positive correlation between falling distance and trauma severity in fall injuries [1]. However, only a few reports have presented details regarding the influence of falling distance on trauma severity, especially among fall injury survivors who were transported to trauma centers. Focusing on this population would exclude patients with minor injuries from falls from a low place and patients who died on site after falling from a high place. Thus, it would be possible to differentiate the characteristics of trauma between survivors of falls from high places and survivors of falls from low places. Understanding the relationship between falling distance and trauma severity of fall injuries would contribute to clarifying the role of orthopedic surgeons in trauma centers. Therefore, this study aims to elucidate the influence of falling distance on the severity of trauma including the number and location of fractures, major organ damage, and length of hospital stay among fall injury survivors who were transported to a trauma center and increase understanding of the characteristics of trauma among survivors of falls from high places from an orthopedic surgeon’s perspective.

## Materials and methods

Subjects

Sixty-five survivors (38 male patients and 27 female patients) of fall injuries who were transported to an emergency trauma center, which is a tertiary emergency medical facility, from May 2014 to October 2016 were included in this study. The mean age was 43.6 years (range: 3-80 years).

Clinical endpoints

We retrospectively reviewed all the 65 patients’ medical records to gather data on the estimated falling distance; the presence of mental illness; whether the fall was a suicide attempt; severe organ damage (head, chest, or abdominal trauma); the number of upper-limb fractures, lower-limb fractures, and spinal vertebral fractures; the severity of thoracolumbar vertebral fracture; the presence of unstable pelvic fracture; the presence of severe nerve injury; the severity of the injury; the duration of intensive care unit (ICU) stay; and the duration of hospitalization. An injury severity score (ISS) was used for evaluating injury severity in the current study [4,5]. ISS was reported to be a potential evaluation tool for the prediction of various injury severity, including mortality, clinical outcomes, and medical cost [4,5]. For evaluating thoracolumbar vertebral fracture severity, we used the McCormack load-sharing classification. As previously reported, the McCormack load-sharing classification consisted of the scoring of three parameters, including comminution, apposition of fragments, and deformity correction, and was used for evaluating spinal anterior column injury and spinal instability [6,7]. Further, in the current study, scores were summed when patients had multiple thoracolumbar vertebral fractures. Severe nerve injury was defined as a Frankel classification of A to C in the current study. Frankel classification evaluated spinal cord function and was used as a tool for spinal cord injury [8,9]. Frankel A showed complete motor loss and sensory loss, and Frankel C showed incomplete motor loss without practical use.

Statistical analysis

All statistical analyses were performed using SPSS version 11.0 (SPSS Inc., Chicago, IL, USA). Correlations between falling distance and the other clinical endpoints were evaluated using the Pearson correlation coefficient. In addition, to evaluate whether injury severity affected ICU stay and hospitalization, correlations between ISS and the duration of ICU stay or hospitalization were evaluated using the Spearman correlation coefficient. The correlation coefficients were classified; r-values of 0.2-0.4, 0.4-0.7, and 0.7-1 were considered weak, moderate, and strong correlations, respectively. To evaluate differences in trauma severity between survivors of falls from high places and survivors of falls from low places, we divided the patients into two groups by fall height: <6 meters (Group L, fall from a low place) and >6 meters (Group H, fall from a high place). Comparisons of all clinical endpoints between the two groups were made using the unpaired t-test or the Mann-Whitney U test and the chi-squared test. Levene’s test was used to assess the equality of variance for variables of interest. For variables with unequal variances, the Mann-Whitney U test was applied. For variables with equal variances, the unpaired t-test was used. Differences with p < 0.05 were considered statistically significant.

## Results

Correlation analysis

**Figure 1:**
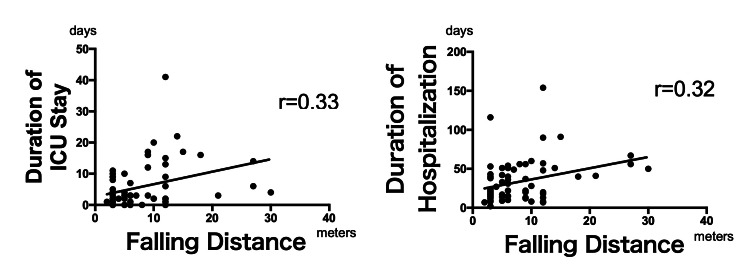
Correlation between falling distance and trauma severity There were significant weak positive correlations between falling distance and the durations of ICU and hospital stay.

There were significant weak positive correlations between falling distance and durations of ICU and hospital stay (ρ = 0.33, 0.32; p < 0.05) (Figure 1). However, there were no significant correlations between falling distance and the other clinical endpoints.

**Figure 2:**
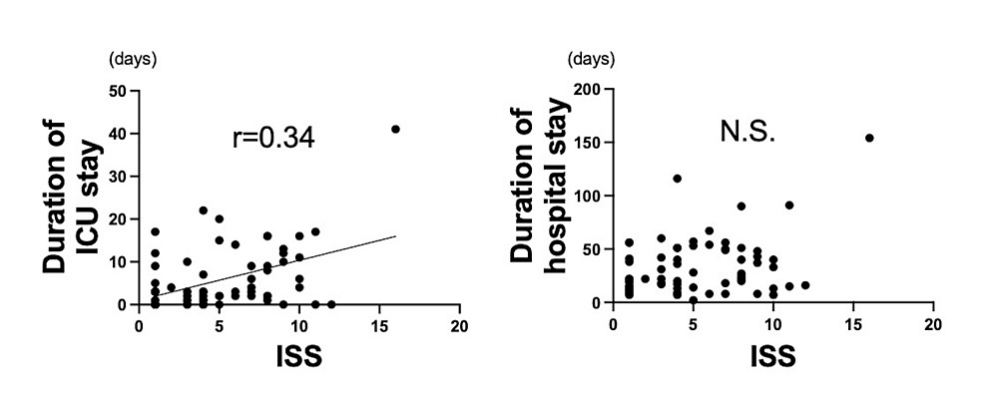
Correlation between injury severity score and durations of ICU or hospital stay There was a significant weak correlation between ISS and duration of ICU stay, but not duration of hospital stay. ISS: injury severity score

On the other hand, there were significant weak positive correlations between ISS and durations of ICU stay (ρ = 0.34; p < 0.05), but not durations of hospitalization (Figure 2).

Comparisons between falls from low places and falls from high places

The mean of falling distances in Group L and Group H was 3.4 meters (range: 2-5 meters) and 11.5 meters (range: 6-30 meters), respectively. The comparisons of each factor between Group L and Group H are summarized in Table 1. There were 28 patients in Group L (22 men and six women) and 37 patients in Group H (16 men and 21 women), and the mean age was 49.8 years in Group L and 39.3 years in Group H. There were significantly more women and younger people in Group H than in Group L (p < 0.05).

**Table 1:** Comparisons between Group L and Group H

	Group L	Group H	p-value
N	28	37	-
Gender (men/women)	22/6	16/21	0.004
Mean age (years)	49.8	39.3	0.020
Presence of mental illness (%)	32.1	78.4	0.000
Presence of suicide attempt (%)	25	75.7	0.000
Presence of head trauma (%)	64.7	23.3	0.069
Presence of chest trauma (%)	27.3	48	0.326
Presence of abdominal trauma (%)	16.7	27.6	0.450
Number of upper-limb fractures (N)	0.36	0.73	0.270
Number of lower-limb fractures (N)	0.68	1.38	0.040
Number of spinal vertebral fractures (N)	0.61	1.14	0.160
Thoracolumbar fracture severity	2.18	5.11	0.038
Presence of unstable pelvic fracture (%)	10.7	24.3	0.161
Presence of severe nerve injury (Frankel A to C) (%)	7.7	8.8	0.885
Injury severity score	5.29	5.08	0.818
Duration of ICU stay (days)	3.11	7.95	0.013
Duration of hospitalization (days)	27.1	39.6	0.046

Regarding psychiatric and psychological factors, the prevalence of mental illness was 32.1% in Group L and 78.4% in Group H, reflecting a statistically significant difference, with a larger percentage in Group H (p < 0.05). Further, the fall was a suicide attempt for 25% of the patients in Group L and for 75.7% of the patients in Group H, reflecting a statistically significant difference, with a higher value in Group H (p < 0.05).

Regarding serious organ damage, head trauma was present in 64.7% of the patients in Group L and in 23.3% of the patients in Group H, chest trauma was present in 27.3% of the patients in Group L and in 48% of the patients in Group H, and abdominal trauma was present in 16.7% of the patients in Group L and in 27.6% of the patients in Group H. There were no statistically significant differences in head, chest, or abdominal trauma between the two fall height groups (p > 0.05) (Table 1).

The details of limb fractures are shown in Table 2. Most of the upper-limb fractures in both groups were distal radius fractures (Table 2). The mean number of upper-limb fractures was 0.36 in Group L and 0.73 in Group H, with no statistically significant difference between the two groups (p > 0.05) (Table 1). Most of the lower-limb fractures in both groups were calcaneus fractures. There were also many femur fractures in Group H (Table 2). The mean number of lower-limb fractures was 0.68 in Group L and 1.38 in Group H, and this difference was statistically significant (p < 0.05) (Table 1).

**Table 2:** Details of limb fractures in Group L and Group H

Upper-limb fracture	Group		Lower-limb fracture	Group	
	L (N = 28)	H (N = 37)		L (N = 28)	H (N = 37)
Proximal humerus	0	1	Proximal femur	2	9
Humeral shaft	0	1	Femoral shaft	0	5
Distal humerus	0	1	Distal femur	1	0
Proximal radius	2	5	Patella	1	1
Radial shaft	0	1	Proximal tibia	1	1
Distal radius	6	8	Tibial shaft	0	3
Proximal ulna	1	1	Distal tibia	2	1
Ulnar shaft	0	2	Fibular	1	4
Distal ulna	0	2	Calcaneus	6	11
Phalange	0	1	Foot	2	8

Most spinal vertebral fractures in both groups were thoracolumbar lesions, and this was especially the case in Group H (Table 3). The average number of spinal vertebral fractures was 0.61 in Group L and 1.14 in Group H, and this difference was not statistically significant (p > 0.05). Thoracolumbar fracture severity as evaluated using McCormack load-sharing classification was 2.18 in Group L and 5.11 in Group H, reflecting a statistically significantly higher value in Group H (p < 0.05).

**Table 3:** Details of spinal vertebral fractures in Group L and Group H

Spinal vertebral fracture	Group	
	L (N = 28)	H (N = 37)
Cervical (C1-7)	3	1
Upper/middle thoracic (T1-9)	8	6
Thoracolumbar (T10-L2)	8	26
Lower lumbar (L3-5)	6	14

Regarding severe nerve injury, two patients in Group L had a Frankel classification of A. In Group H, one patient had a Frankel classification of B, and two had a Frankel classification of C. The prevalence of severe nerve injury was 7.7% in Group L and 8.8% in Group H, with no statistically significant difference between the two groups (p > 0.05) (Table 1). The prevalence of unstable pelvic fracture was 10.7% in Group L and 24.3% in Group H. Although there were more cases of unstable pelvic fracture in Group H than in Group L, this difference was not statistically significant (p > 0.05) (Table 1).

The mean ISS was 5.29 in Group L and 5.08 in Group H, with no statistically significant difference between the two groups (p > 0.05). The mean duration of ICU stay was 3.11 days in Group L and 7.96 days in Group H, and this difference was statistically significant (p < 0.05). The mean duration of hospital stay was 27.1 days in Group L and 39.6 days in Group H, which represents a statistically significantly longer duration in Group H (p < 0.05) (Table 1).

## Discussion

In this study, falls from high places were more often suicide attempts than were falls from low places. Further, compared with falls from low places, falls from high places resulted in more lower-limb fractures and spinal vertebral fractures, more severe thoracolumbar fractures, and longer durations of ICU and hospital stay. In addition, higher ISS was associated with longer durations of ICU stay, but not hospitalization.

Regarding the relationship between falling distance and the severity of trauma in fall injuries, Maull et al. reported that, for high-velocity deceleration injuries, injury severity increased when the rate of deceleration was higher and when the distance through which the body was decelerated decreased [10]. Frederic et al. also reported that falling distance was a predictive factor for mortality from fall injuries [1]. Kusior et al. concluded that understanding the relationships between injuries and fall height and observing the overall number of injuries seen in patients who fall from a height may be useful for inferring the height of a fall [2]. These findings indicated that falling distance was a potential risk factor of injury severity.

Several authors reported, in their study of victims of fall injury, falling distance was significantly correlated with ISS or chest injury [11,12]. By contrast, in the current study, ISS and the presence of chest injury did not show any significant differences between high place fall and low place fall. One of the reasons for this discrepancy between the current study and the previous reports would be the differences of subjects such as survivors and victims. Few studies have evaluated the influence of falling distance on specific aspects of trauma severity, especially among fall injury survivors who were transported to trauma centers. Our study showed that, among survivors of fall injuries, falling distance was associated with the number of lower-limb fractures, the severity of spinal vertebral fractures, and the duration of ICU and hospital stays. Mekkodathil et al. reported in their study of 4,040 patients with fall injury that 29% of patients with fall injury had a spinal injury, and the spine was one of the most common injury sites in fall injury [13]. These findings indicated that falls especially from high places resulted in vertebral fracture severity, more lower-limb fractures, and longer ICU and hospital stays. Therefore, from the perspective of orthopedic surgeons, especially spinal and trauma surgeons, early intervention including surgery for vertebral fractures and lower-limb fractures is needed among survivors of falls from high places.

Regarding the factors predicting fall injury severity other than falling distance, several authors reported that falling posture, defensive reactions, falling trajectory, and patient characteristics, including age and weight, were potential predicting factors of injury severity [1,11,14-16]. Frederic et al. reported that patient age, the ground characteristics where the fall occurs, and falling posture determined mortality from fall injuries [1]. Conversely, another study reported that the characteristics of the ground where the fall occurs had no effect on trauma severity [17]. In terms of falling posture, Goonetilleke et al. identified three falling patterns: feet-first, head-first, and side-first [18]. According to the study conducted by Teh et al., compared with other types of landings, feet-first landings decreased the falling energy reaching important organs, including the head, chest, and abdomen; therefore, falling feet first might lead to less severe organ damage compared with other falling postures [19]. In the current study, more patients had attempted suicide in the group surviving falls from high places than in the group surviving falls from low places. Feet-first landings may be more common among patients who attempt suicide [20]. Therefore, our findings indicate that whether there is severe organ damage and upper-limb fractures might depend on the falling posture, regardless of falling distance.

Regarding the relationships between injury severity and clinical outcomes, in this study, higher ISS was associated with longer durations of ICU stay, but not hospitalization. As mentioned above, ISS was firstly described by Baker et al. and reported to be used as a potential prediction tool for mortality and clinical outcomes [4,5]. Several authors reported that ISS were correlated with durations of ICU stay and hospitalization [21,22]. In the current study, fall injury survivors who had multiple severe organ injuries, including head, chest, and abdominal injury, had a longer duration of ICU stay. However, fall injury survivors who also had multiple fractures, especially at the spine and lower limbs, might also have longer hospitalization because of limitations of rehabilitation, such as delay of weight-bearing, independent of the presence of severe organ injury. Therefore, early intervention for spinal and lower limb fractures might be needed for shortening the hospitalization.

There were some limitations in this study. First, because the current study was a retrospective study, we could not review the properties of the ground where the fall occurred. Second, conducting the study in an emergency trauma center introduced the potential biases of excluding patients with minor injuries after falling from low places, as well as those who died on site after falling from high places. Third, regarding the definition of falls from a high place, we defined falls from >6 meters as falls from a high place in the current study. As mentioned above in the Introduction section, this definition was based on the guidelines for field triage of injured patients. However, as discussed above, several factors, including falling posture and defensive reactions, are associated with injury severity. Moreover, there would not be sufficient evidence about grouping by falling distance. Therefore, further study would be necessary.

## Conclusions

We found that there were more suicide attempts among patients surviving falls from high places than among those surviving falls from low places. Further, compared with falls from low places, falls from high places resulted in more lower-limb fractures, higher McCormack load-sharing classification scores, and longer ICU and hospital stays. These findings indicate that falling distance may affect lower-limb and vertebral fractures and may lead to long ICU and hospital stays. From an orthopedic surgeon’s perspective, early intervention including surgery for vertebral fractures and lower-limb fractures is needed among fall injury survivors who are transported to trauma centers.
